# Prevalence of kidney damage in Chinese elderly: a large-scale population-based study

**DOI:** 10.1186/s12882-019-1525-5

**Published:** 2019-09-02

**Authors:** Honglan Wei, Yaqiong Yan, Jie Gong, Junwu Dong

**Affiliations:** 1 Department of Nephrology, Wuhan Fourth Hospital, Puai Hospital, Tongji Medical College, Huazhong University of Science and Technology, Wuhan, Hubei 430033 People’s Republic of China; 2 Wuhan Center for Disease Control and Prevention, Wuhan, Hubei 430000 People’s Republic of China

**Keywords:** Kidney damage, Epidemiology, Chinese elderly

## Abstract

**Background:**

In China, both population aging and kidney damage has become emerging public health challenges. Despite the number of elders is huge, data on kidney damage in this population are scarce. The present study aimed to describe the prevalence of kidney damage among older adults in Wuhan, China.

**Methods:**

To describe the prevalence of kidney damage among Chinese elderly, the health screening data of 350,881 adults older than 65 years in Wuhan, China were collected and analyzed. Kidney damage was defined as eGFR less than 60 mL/min per 1·73 m^2^ or the presence of proteinuria. Decreased renal function was defined as an eGFR < 60 mL/min/1.73 m^2^. Proteinuria was defined as urine protein ≥1+ and without urine WBC or nitrite positive. The associated risk factors of eGFR decline and kidney damage were analyzed by multivariate logistic regression.

**Results:**

The age-standardized prevalence of kidney damage, decreased renal function and proteinuria was 17.2, 13.5 and 5.3%. Among the patients, up to 74.4% was stage 3. The prevalence of kidney damage and eGFR decline were higher in suburbs than in urban (18.3% vs 16.0 and 14.6% vs 12.4%). Factors independently associated with kidney damage were age, female, BMI, abdominal circumference, hypertension, diabetes, stroke and coronary heart disease.

**Conclusions:**

Kidney damage has become an important public health problem in Chinese elderly. More attention should be paid to elderly lived in suburbs or rural area in our further work.

## Background

Population aging is an emerging worldwide public health challenge in the twenty-first century, which is especially prominent in low- and middle-income countries [1]. It is estimated that over 1.5 billion individuals worldwide will be 65 years or older by 2050 [1]. China is the world’s most populous country and the largest developing country, in which population aging is particularly serious. China only used 18 years (1981–1999) to enter the aging society and the rate of aging is accelerating. According to the sixth national census in 2010, 118.8 million Chinese were 65 years or older, accounting for 8.87% of the total Chinese population. The proportion rose 1.91 points than 10 years ago and was expected increase to 16.23% of the population in 2030 [2].

Aging is associated with an increased prevalence of chronic diseases, such as diabetes, hypertension, heart disease and stroke. Patients with these chronic diseases suffer from chronic kidney disease (CKD) risk multiplier. Except that, age is an independent risk factor of CKD [3]. In developing countries, CKD is highly prevalent [4]. Evidently, CKD, as a major global public health problem over the last decade, is especially prevalent in the elderly population in developing countries [5]. The costs of care at the elderly patients with CKD were huge [6].

Delayed diagnosis of CKD in the elderly potentially lead to further damage and increase the risk of death or disability [7]. Early identification and treatment of older individuals with CKD are needed worldwide [7]. The incidence of acute kidney injury (AKI) is also higher in older CKD than in young people [8]. In China, despite the number of elders is huge, data on kidney damage (whether CKD or AKI) in this population are scarce. To our knowledge, there was no large-scale population-based studies described the prevalence of kidney damage in Chinese older adults. The present study aimed to describe the prevalence of kidney damage among older adults in Wuhan, China.

## Methods

### Setting and participants

The design of this study was a population-based study of kidney damage among individuals older than 65 years in Wuhan, China. Wuhan consists of 17 districts and has 854, 833 elderly. Under a scheme launched by Wuhan municipal government, Wuhan Center for Disease Control and Prevention provided health screening for 372,990 older adults in 11 urban districts and 6 suburban districts from May 2012 to March 2013. The participants were voluntary and informed consent. The health screening included health status questionnaire (eg, age, sex, education, health history, smoking and alcohol consumption), blood pressure, height, weight, waist circumference and core laboratory tests, containing blood tests, urinalysis, liver function, kidney function, fasting plasma glucose (FPG), blood lipids, abdominal B ultrasound, ECG and chest X-ray. All blood and urine samples were analyzed at the central laboratory, which successfully completed a standardization and certification program. The individuals with abnormal physical or laboratory tests at screening were recommended to the related departments to review and treat in the general hospital.

### Screening protocol and assessment criteria

#### Proteinuria

Urinary protein was measured from a fresh morning spot urine sample with the Automatic Urine Analyzer (Roche Diagnostics, Germany). The result was reported as negative, trace, +, ++, +++ or ++++. Proteinuria was defined as urine protein ≥1+ and without urine WBC or nitrite positive.

#### eGFR

To measure the estimated glomerular filtration rate (eGFR), blood was collected by venepuncture after an overnight fast of at least 10 h. Serum creatinine was measured with Jaffe’s kinetic. The Modification of Diet in Renal Disease (MDRD) equation for Chinese CKD patients was used to calculate the eGFR [9].

eGFR (mL/min/1.73 m^2^) = 175 × Scr(mg/dL)^-1.24^ × age(year)^-0.179^[female× 0.79].

Decreased renal function was defined as an eGFR < 60 mL/min/1.73 m^2^. Kidney damage was defined as eGFR less than 60 mL/min per 1·73 m^2^ or the presence of proteinuria. Renal function was classified based on eGFR as follows: G1 (eGFR≥90 ml/min/1.73 m^2^), G2 (eGFR, 60–89 ml/min/1.73 m^2^),G3(eGFR, 30–59 ml/min/1.73 m^2^), G4 (eGFR,15–29 ml/min/1.73 m^2^), G5 (eGFR< 15 ml/min/1.73 m2).

#### Hypertension

All participants were measured the blood pressure by a mercury sphygmomanometer after quiet rest for at least 15 min. Systolic blood pressure (BP) ≥ 140 mmHg or diastolic BP ≥ 90 mmHg for two times, or with a history of hypertension and use of antihypertensive medication was defined as hypertension.

#### Diabetes

The fasting plasma glucose≥7 mmol/L, and/or 2-h postprandial plasma glucose≥11.1 mmol/L, or with the history of diabetes was defined as diabetes.

#### Hyperlipidemia

Hyperlipidemia was defined as total cholesterol≥6.22 mmol/L and (or) triglycerides ≥2.26 mmol/L.

#### Obesity

Obesity was defined as body mass index (BMI) > 28, and central obesity was defined as a waist measurement > 85 cm for men or > 80 cm for women.

### Statistical analysis

Data management and analyses were performed on SAS software, version 9.13 (SAS Institute, Cary, NC. USA). Continuous data were presented as mean ± SD. Mean comparison was performed by t test. The age-standardized prevalence of kidney damage, decreased renal function and proteinuria were calculated using the direct standardization method. The standardized population is based on the population composition of the 6th National Census in 2010. The data comes from the National Bureau of Statistics of the People’s Republic of China. The ratio was compared by Chi-square test. The associated risk factors of eGFR decline and kidney damage was analyzed by multivariate logistic regression, and *P*-value less than 0.05 was taken as the threshold for statistical significance.

## Results

A total of 372,990 elders participated in the examination. 350, 881(94.07%) cases with complete data were included for the current study, and the average age was 71.9 ± 5.6 years. 163,454 (46.58%) were male and 187,427 (53.42%) were female. 172,028 (49.03%) lived in urban districts and 178,853(50.97%) were from suburbs. The average age of urban participants was 72.2 ± 5.6 years, and the participants from suburban areas were 71.6 ± 5.6 years. Table 1 shows the distribution of age and sex in different districts. In total, 144,377 were 65 to 69 years, accounting for 41.1%. 29.1% of the participants were 70 to 74 years and 19.5% were 75 to 79 years. 10.3% were 80 years or older. The clinical characteristics of the study population are shown in Table 2.

**Table 1:** Age and sex distribution of study population

	Sex	Age, n (%)				Total
		65~	70~	75~	80~	
Urban	Male	29,454 (37.7)	22,499 (28.8)	17,276 (22.1)	8871 (11.4)	78,100 (45.4)
	Female	36,417 (38.8)	27,798 (29.6)	20,170 (21.5)	9543 (10.1)	93,928 (54.6)
	Total	65,871 (38.3)	50,297 (29.2)	37,446 (21.8)	18,414 (10.7)	172,028 (49.0)
Suburbs	Male	37,399 (43.8)	25,645 (30.0)	15,001 (17.6)	7309 (8.6)	85,354 (47.7)
	Female	41,107 (44.0)	26,168 (28.0)	15,845 (16.9)	10,379 (11.1)	93,499 (52.3)
	Total	78,506 (43.9)	51,813 (29.0)	30,846 (17.2)	17,688 (9.9)	178,853 (51.0)
Total	Male	66,853 (40.9)	48,144 (29.5)	32,277 (19.7)	16,180 (9.9)	163,454 (46.6)
	Female	77,524 (41.4)	53,966 (28.8)	36,015 (19.2)	19,922 (10.6)	187,427 (53.4)
	Total	144,377 (41.1)	102,110 (29.1)	68,292 (19.5)	36,102 (10.3)	350,881 (100.0)

**Table 2:** Clinical characteristics of study population

	Male	Female	Total
Systolic blood pressure (mmHg)	134.5 ± 20.4*	135.7 ± 21.0	135.1 ± 20.7
Diastolic blood pressure (mmHg)	80.9 ± 11.3*	79.9 ± 11.1	80.4 ± 11.2
Blood urea nitrogen (mmol/L)	6.1 ± 2.3*	5.7 ± 2.3	5.9 ± 2.3
Serum creatinine (μmol/L)	90.4 ± 26.9*	74.7 ± 23.2	82.0 ± 26.2
Blood cholesterol (mmol/L)	4.7 ± 1.1*	5.1 ± 1.1	4.9 ± 1.1
Serum triglyceride (mmol/L)	1.0 (0.7–1.5)	1.3 (0.9–1.8)	1.2 (0.8–1.7)
Fast blood glucose (mmol/L)	5.5 ± 1.4*	5.5 ± 1.5	5.5 ± 1.5
BMI	23.0 ± 3.3*	23.4 ± 3.6	23.2 ± 3.5
Abdominal circumference (cm)	83.5 ± 8.9*	81.7 ± 9.0	82.5 ± 9.0
Diabetes(n, %)	18,623 (11.5)*	24,923 (13.4)	43,546 (12.5)
Hypertension(n, %)	95,560 (58.9)*	114,831 (61.8)	210,391 (60.5)
Hyperlipidaemia(n, %)	27,361 (16.8)*	51,573 (27.6)	78,934 (22.6)
Obesity(n, %)	11,011 (6.7)*	18,734 (10.0)	29,745 (8.5)
Coronary heart disease(n, %)	11,154 (6.9)*	15,086 (8.1)	26,240 (7.5)
Stroke(n, %)	6377 (3.9)*	6135 (3.3)	12,512 (3.6)

*P < 0.05

### Prevalence of decreased renal function

In urban, 11.7% males and 11.4% females had decreased renal function (*p* = 0.059). In suburbs, the proportion of eGFR< 60 mL/min/1.73 m^2^ was 11.0% in males, which was lower than in females (15.7%) (*p* = 0.000). In total, 11.4% male participants and 13.5% female participants had decreased renal function (*p* = 0.000). The eGFR of 43,949 participants was lower than 60 mL/min/1.73 m^2^, accounting for 12.5%. The proportion of decreased renal function in suburbs participants was higher than in urban participants (13.4% versus 11.6%, *p* = 0.000) (Table 3). To research the prevalence of renal function decline in different age groups, the participants were divided into groups by 5-year ages. The percentages of G3 and G4 were increased with age both in urban and suburbs (*p* = 0.000). In urban, 0.2% was G5 in participants aged 80 years or older and in other groups was only 0.1%. In suburbs, the percentages of G5 had no difference in various groups. After adjusted for age, 11.8% participants from urban were G3, which was lower than in suburbs (14.0%, *p* = 0.000). There was no difference of G4 and G5 percentages in urban and suburbs after adjusted for age. In total, 12.9% of participants was G3, 0.5% G4 and 0.1% G5 after adjusted for age (*p* = 0.000). The age-standardized prevalence of decreased renal function in urban was 12.4%, in suburbs was 14.6 and in total was 13.5%. (Table 4).

**Table 3:** The percentages of decreased renal function in urban and suburbs

	Sex	Grades, n (%)			Total
		G3	G4	G5	
Urban	Male	8698 (11.2)	382 (0.5)	77 (0.1)	9157 (11.7)
	Female	10,217 (10.9)	419 (0.5)	102 (0.1)	10,738 (11.5)
	Total	18,915 (11.0)	801 (0.5)	179 (0.1)	19,895 (11.6)
Suburbs	Male	8939 (10.5)	369 (0.4)	63 (0.1)	9371 (11.0)
	Female	14,227 (15.2)	375 (0.4)	81 (0.1)	14,683 (15.7) *
	Total	23,166 (12.9)	744 (0.4)	144 (0.1)	24,054 (13.4) *
Total	Male	17,637 (10.8)	751 (0.5)	140 (0.1)	18,528 (11.4)
	Female	24,444 (13.0)	794 (0.4)	183 (0.1)	25,421 (13.5) *
	Total	42,081 (12.0)	1545 (0.4)	323 (0.1)	43,949 (12.5)

*P < 0.05

**Table 4:** The percentages of renal function decline in different age groups

	Crude, n (%)				Adjusted(95%CI)
	65~	70~	75~	80~	
Urban					
G3	4247 (6.5)	5266 (10.5)	5508 (14.7)	3894 (21.2)	11.8 (11.7–11.9)
G4	154 (0.2)	203 (0.4)	213 (0.6)	231 (1.3)	0.5 (0.48–0.52)
G5	52 (0.1)	54 (0.1)	42 (0.1)	31 (0.2)	0.1 (0.09–0.11)
Suburbs					
G3	7631 (9.7)	6564 (12.7)	5294 (17.2)	3677 (20.8)	14.0 (13.9–14.1) *
G4	196 (0.3)	211 (0.4)	182 (0.6)	155 (0.9)	0.5 (0.48–0.52)
G5	50 (0.1)	44 (0.1)	30 (0.1)	20 (0.1)	0.1 (0.09–0.11)
Total					
G3	11,878 (8.2)	11,830 (11.6)	10,802 (15.8)	7571 (21.0)	12.9 (12.8–13.0) *
G4	350 (0.2)	414 (0.4)	395 (0.6)	386 (1.1)	0.5 (0.48–0.52)
G5	102 (0.1)	98 (0.1)	72 (0.1)	51 (0.1)	0.1 (0.09–0.11)

*P < 0.05

### Prevalence of proteinuria

**Fig. 1:**
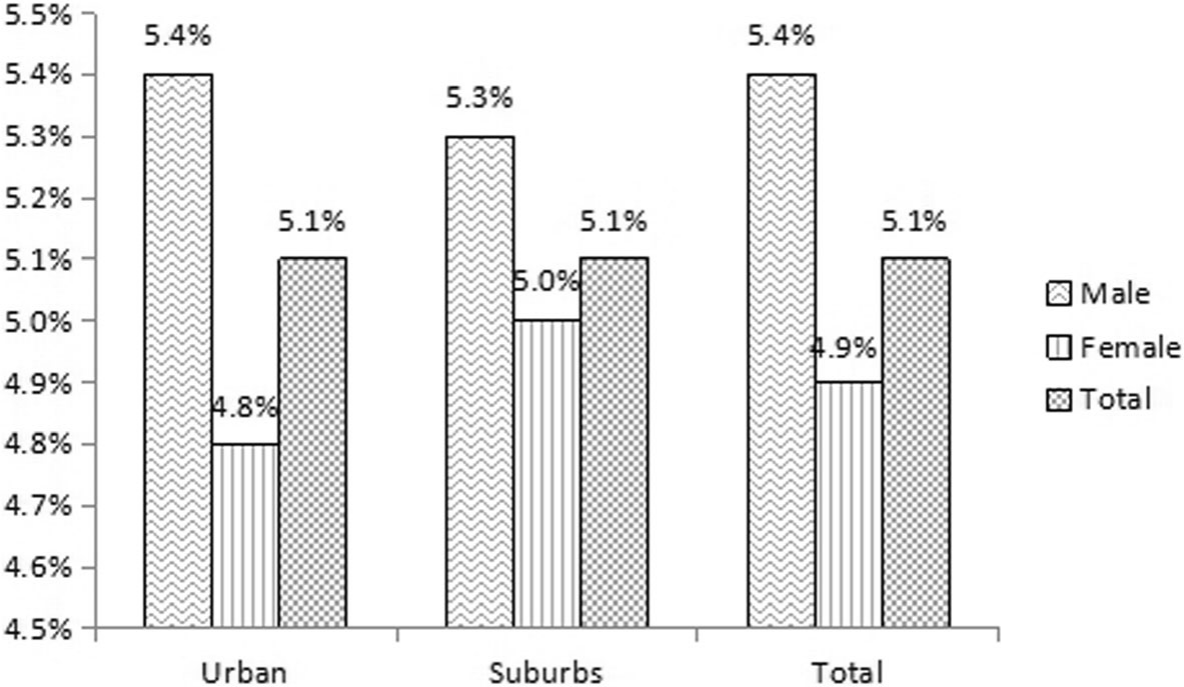
Prevalence of proteinuria by sex in different districts

In urban, 4, 230 males had proteinuria, accounting for 5.4%, which was higher than in females (4.8%, *p* = 0.000). In suburbs, 4539 (5.3%) males and 4629(5.0%) females had proteinuria (*p* = 0.000). In all participants, the percentage of proteinuria in males was 5.4%, which was higher than in females (4.9%, *p* = 0.000). In total, 17,858 (5.1%) participants had proteinuria and the prevalence of proteinuria in urban and suburbs was the same (Table 5 and Fig. 1). After adjusted for age, the percentage of proteinuria was 5.3% in all participants.

**Table 5:** Prevalence of decreased renal function, proteinuria and kidney damage

	eGFR< 60 mL/min/1.73 m^2^			Proteinuria			Kidney damage		
	Total (n)	Positive (n)	Prevalence (%)	Total (n)	Positive (n)	Prevalence (%)	Total (n)	Positive (n)	Prevalence (%)
Urban	172,088	19,895	11.6	172,088	8690	5.1	172,088	25,933	15.1
Suburbs	178,853	24,054	13.4*	178,853	9168	5.1	178,853	30,610	17.1*
Total	350,881	43,949	12.5	350,881	17,858	5.1	350,881	56,543	16.1

*P < 0.05

**Fig. 2:**
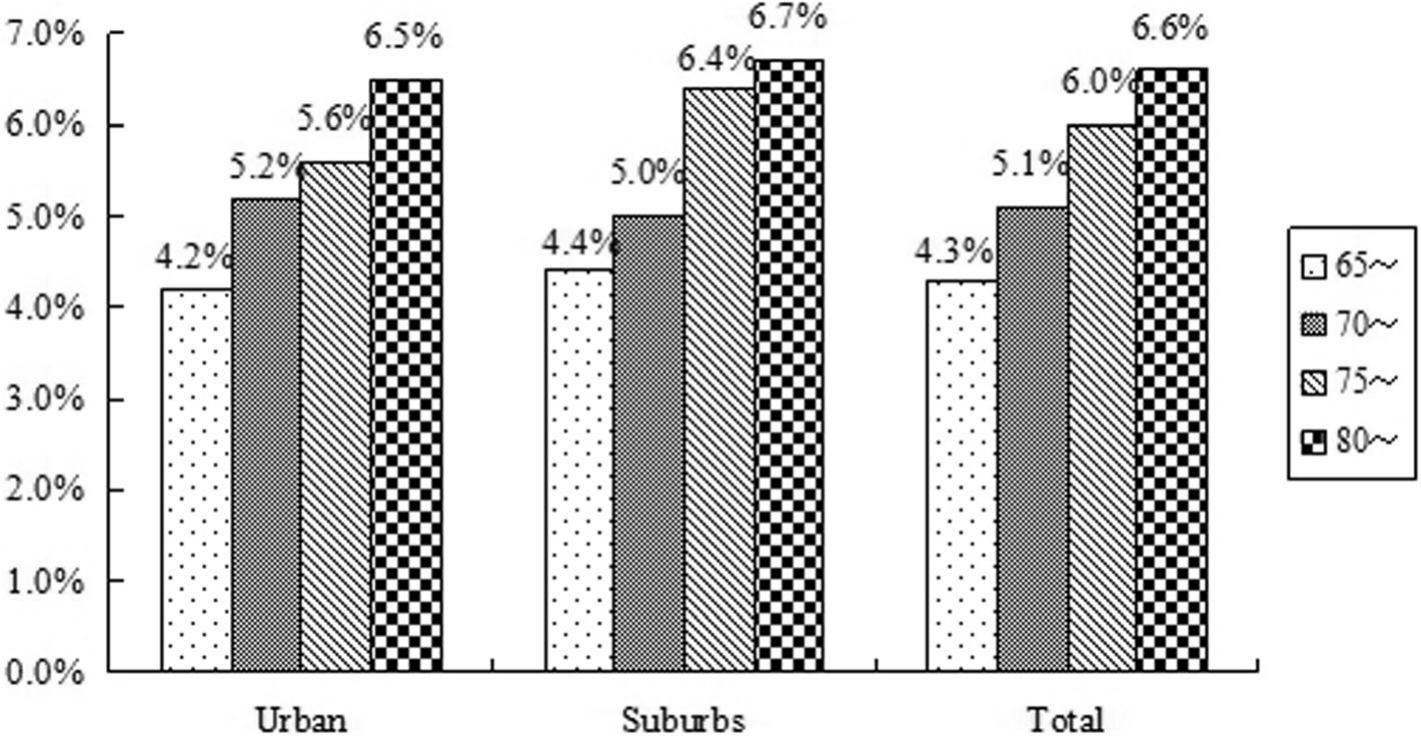
Prevalence of proteinuria by 5-year age groups in different districts

The prevalence of proteinuria was increased with age both in urban and suburbs. In group of age 70~74 years, 5.2% participants from urban had proteinuria, which was higher than in suburbs (*p* = 0.000). In other groups, the prevalence of proteinuria was higher in suburbs than in urban (*p* = 0.000, Fig. 2). After adjusted with age, the percentage of proteinuria in suburbs was 5.4%, which was 5.2% in urban (*p* = 0.000).

### Prevalence of kidney damage

Twelve thousand fifty-seven males from urban had kidney damage, accounting for 15.4%, which was higher than in suburbs (14.9%, *p* = 0.002). The prevalence of kidney damage in females from urban was 14.8%, which was lower than in suburbs (19.2%, *p* = 0.000). In total, 15.2% males and 17.0% females had kidney damage (*p*  = 0.000). The prevalence of kidney damage in urban was 15.1%, which was lower than in suburbs (17.1%, *p* = 0.000) (Table 5 and Table 6). Fifty-six thousand five hundred forty-three (16.1%) participants had kidney damage, and the percentage was 17.2% after adjusted for age (Table 5).

**Table 6:** Prevalence of kidney damage by sex in different districts

	Crude, n (%)			Adjusted for age (%)(95%CI)
	Male	Female	Total	
Urban	12,057 (15.4) *	13,876 (14.8)	25,933 (15.1)	16.0 (15.9–16.1)
Suburbs	12,708 (14.9)	17,902 (19.2) *	30,610 (17.1) *	18.3 (18.2–18.4) *
Total	24,765 (15.2)	31,778 (17.0) *	56,543 (16.1)	17.2 (17.1–17.3)

*P < 0.05

**Fig. 3:**
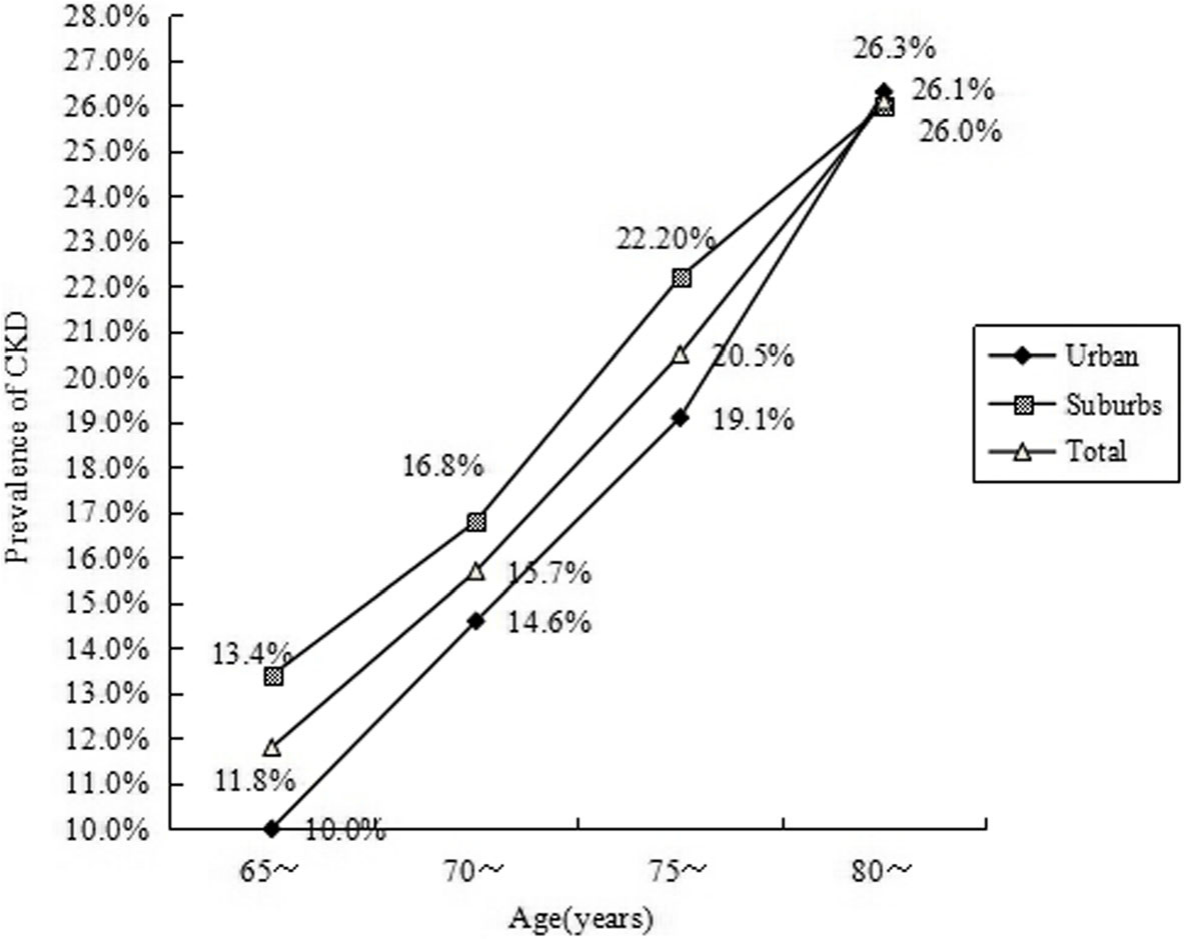
Prevalence of kidney damage by 5-year age groups in elders

In the group of older than 80 years, 26.3% participants from urban had kidney damage, which was 26.0% in suburbs (*p* = 0.517). But in the groups of ages 65–69 years, 70-74 years and 75-79 years, the kidney damage prevalence in urban was lower than in suburbs (*p* = 0.000). In total, 11.8% participants ages 65–69 years, 15.7% participants ages 70–74 years, 20.5% participants ages 75–79 years and 26.1% participants ages 80 years or older had kidney damage (*p* = 0.000, Fig. 3).

In total, there were 56,543 participants had kidney damage. Among the kidney damage participants, 38,685 (68.4%) patients had eGFR decline, but without proteinuria; 12,594 (22.3%) patients with proteinuria and normal renal function; only 5264 (9.3%) patients had both proteinuria and decreased renal function. 42,081 (74.4%) was stage 3, 1545 (2.7%) stage 4 and only 323 (0.6%) was stage 5 .

### Associated risk factors of eGFR decline and kidney damage

Age, sex, blood urea nitrogen, serum creatinine, blood cholesterol, serum triglyceride, fast blood glucose, BMI, abdominal circumference, history of hypertension, diabetes, hyperlipidaemia, obesity, stroke and coronary heart disease were analyzed by the multivariate logistic regression and age, female, BMI, abdominal circumference, history of hypertension, diabetes, stroke and coronary heart disease were all independently associated with eGFR decline and kidney damage (Table 7).

**Table 7:** Factors associated with kidney damage

	eGFR< 60 ml/min/1.73m^2^		Kidney damage	
	OR	95%CI	OR	95%CI
Age	1.422	1.374–1.473	1.069	1.044–1.095
Female	1.092	1.071–1.12	1.099	1.079–1.120
BMI	1.082	1.068–1.096	1.080	1.067–1.094
Abdominal circumference	1.112	1.090–1.134	1.108	1.086–1.130
Hypertension	1.575	1.543–1.607	1.577	1.546–1.609
Diabetes	1.614	1.575–1.654	1.626	1.587–1.667
Stroke	1.276	1.221–1.334	1.275	1.220–1.332
Coronary heart disease	1.243	1.204–1.283	1.239	1.201–1.279

## Discussion

Although much is known about kidney damage in elderly, a great deal remains to be researched. To our knowledge, this study is the first large-scale population-based study to research the prevalence of kidney damage in Chinese elderly. In this study population, the age-standardized prevalence of kidney damage, decreased renal function and proteinuria was 17.2, 13.5 and 5.3%. Based on these results, 20.4 million Chinese aged 65 years or older have kidney damage, and 16.0 million have decreased renal function. Several studies have reported the high prevalence of chronic kidney disease in Chinese [3, 10–14]. However, fewer large-scale population-based studies focused on Chinese elderly [15]. We used the same standard to define urine protein and low eGFR as used in a large study from Taiwan, which showed 37.2% participants aged 65 years or older had CKD [10]. It is more than two times higher than our results. In the United States, the prevalence of CKD among older adults ranges from 25.9 to 44% [16–18]. In Canada, 31 to 35.7% elderly had CKD when the eGFR was estimated by the MDRD equation [19–22]. In Northern Senegal, the prevalence of CKD in elderly was 14.3%, which was lower than our results [23].

In this study, we found 15.2% males and 17.0% females had kidney damage. It suggested that the prevalence of kidney damage was gender-different and females had a higher prevalence of kidney damage than males. The same phenomenon was revealed in some other large studies [24]. In Canada, Garg et al. reported 27.1% males and 38.8% females aged ≥65 years had CKD [20]; Hemmelgarn et al. found 32% males and 38.2% females aged ≥66 years had CKD [21]. In Switzerland, the prevalence of CKD in male and female participants older than 65 years was 12.9 and 35.9% when the eGFR was estimated by the MDRD equation [25]. In Australian aged 65 years or older, 51.8% males and 57.2% females had CKD when the eGFR was estimated with CG equation [26]. The researchers from Japan and Iceland also found the females elderly had a higher prevalence of CKD than males [27, 28], but the results of study from Italy was converse [29]. The mechanism of gender-different prevalence of CKD is still unclear, which may be associated with the difference between females and males in glomerular structure, glomerular haemodynamics, and the hormone metabolism [30].

Our results revealed the prevalence of decreased renal function, proteinuria increased in parallel with age, which was consistent with the results from Australia [26]. The studies from SardiNIA [31], Canada [20], USA [32], Italy [29], Norway [33] and Iceland [27] also reported that the prevalence of CKD was rising with increasing age.

Among the kidney damage participants, proteinuric kidney damage only accounted for 22.3%, but up to 74.4% patients was stage 3. In Canada elderly, the percentage of CKD stage 3 was higher than CKD stage 4–5 in any age groups (by 5-years) and in any sex [20]. In Australia, up to 53.1% participants aged 65 years or older was CKD stage 3, but only 1.7% elderly had eGFR< 30 mL/min/1.73 m^2^ [26]. It suggested that the elderly with CKD mostly are CKD stage 3. Whether these patients’ renal function is persistently stable or rapid progress requires further observation. Focusing attention on elderly people with CKD stage 3 may prove to be a more cost-effective approach to preventing ESRD in elderly, although further work is required to confirm this hypothesis [34].

The data from the current study suggest that the prevalence of proteinuria in urban and suburbs residents was the same, but the prevalence of decreased renal function in suburbs residents was higher than in urban residents. The prevalence of kidney damage in suburbs residents was higher than in urban residents in any age groups. Zhang LX et al. reported that more rural residents had CKD than urban residents in Chinese general population (average age 49.6 ± 15.2 years) [3]. The result was consistent with ours. But in their study, fewer rural residents had eGFR decline than urban residents, but more had albuminuria [3]. It may because their participants were younger than ours and the prevalence of eGFR decline was obviously lower than albuminuria (1.7% vs. 9.4%) [3]. In China, suburbs and rural residents are more to farm for a living. They often come into contact with pesticides at work or in life. Some study has reported that phosphate fertilizer is a main source of arsenic in areas affected with chronic kidney disease of unknown etiology in Sri Lanka [35]. Except that, for suburbs and rural residents, Medicare amount paid by the government is lower than urban residents. Therefore, fewer suburbs and rural residents can have health care examination every year. Then, the diseases without special clinical symptoms, such as CKD, always are discovered late. To treat the end-stage renal disease, the expense will be much higher than treating early kidney disease. The government and population will bear a heavier financial burden, which will result in a vicious cycle in suburbs and rural areas [6]. It suggests that focusing attention on elderly suburbs and rural residents may reduce the cost of kidney diseases effectively.

### Strengths and limitations of the study

The present study is the first large-scale population-based study to research the prevalence of kidney damage in Chinese elderly. But it had several limitations. First, uric protein was only detected by dipstick urinalysis, which has less favorable diagnostic properties than urine albumin-creatinine ratio (ACR) for the assessment of proteinuria [36]. To research the relationship between kidney function, proteinuria and adverse outcomes, Hemmelgarn BR et al. assessed proteinuria by urine dipstick or ACR. Their results suggested that dipstick urinalysis can provide reliable prognostic information and the magnitude of excess risk observed with heavy proteinuria appeared similar whether assessed by dipstick or by ACR [37]. The research showed that both sensitivity and specificity of dipstick proteinuria were > 80% in older patients. Dongmin Lim et al. suggested that urine dipstick test can be used for screening in older outpatients with ACR ≥300 mg/g. However, they cannot recommend the test as a screening tool with ACR ≥30 mg/g as the reference owing to its low sensitivity [38]. Except that, ACR is much more expensive than dipstick urinalysis. The researchers from the USA which followed up the costs in a cohort of patients older than 50 years, the results showed that targeted annual microalbuminuria screening, relative to no screening, cost effective only in people with diabetes, hypertension, or both [39]. Second, the eGFR decline is associated with the age, the eGFR decline in elderly whether correlated with the diseases progress is needed to further research. Third, eGFR was not directly measured in the study population. We only used the simplified Chinese MDRD Study equation to estimate eGFR, which may over- or underestimate the actual eGFR in the elderly. A study from German reported that the prevalence of CKD in the elderly was very variable based on the used estimating equation, which showed that prevalence of CKD (eGFR < 60 mL/min/1.73 m2) was 34.3% by MDRD, 33.0% by CKD-EPI, and 14.6% by the CysC-based eGFR [19]. Further study will be needed to explore an equation which is more suitable for estimating eGFR of Chinese elderly. Fourth, this article is not a cross-sectional survey. The participants based on voluntary consent, which may select less healthy people, such as hypertension or diabetes. In 2010, The 3rd Chronic Non-communicable Disease & Risk Factor Surveillance in China was conducted in 31 provinces and Xinjiang Production & Construction Corps, which showed the prevalence of hypertension and diabetes in Chinese adults aged > = 60 years were 66.9 and 19.6% [40]. In our participants, they were 60.5 and 12.5%. So we think the selection bias can be rule out.

## Conclusions

Our results show that kidney damage has become an important public health problem in Chinese elderly. Among the older patients, stage 3 is prominent and special attention should be paid to which in our further work. We also found that decreased renal function in suburbs residents was higher than in urban residents, improve the income and medicare amount of which in future may alleviate the social financial burden effectively.

## Data Availability

All data that support the conclusions of this manuscript are included within the article.

## Abbreviations

95% CI: 95% confidence interval
ACR: Albumin-creatinine ratio
AKI: Acute kidney diseas
BMI: Body mass index
BP: Blood pressure
CKD: Chronic kidney disease
eGFR: Estimated glomerular filtration rate
ESRD: End-stage renal disease
FPG: Fasting plasma glucose
MDRD: Modification of Diet in Renal Disease

## References

[1] Ganguly S. Good health adds life to years. J Indian Med Assoc. 2012;110(4):212–3. 23025218

[2] Zhang KD, Guo P (2010). Chinese population aging and the elderly status of blue book.

[3] Zhang LX, Wang F, Wang L, Wang W, Liu BC, Liu J, Chen MH, He Q, Liao YH, Yu XQ, Chen N, Zhang J, Hu Z, Liu HY, Hong DQ, Ma LJ, Liu H, Zhou XL, Chen JH, Pan L, Chen W, Wang WM, Li XM, Wang HY (2012). Prevalence of chronic kidney disease in China: a cross-sectional survey. Lance..

[4] Nugent RA, Fathima SF, Feigl AB, Chyung D (2011). The burden of chronic kidney disease on developing nations: a 21st century challenge in global health. Nephron Clin Pract.

[5] Glassock RJ, Warnock DG, Delanaye P (2017). The global burden of chronic kidney disease: estimates, variability and pitfalls. Nat Rev Nephrol.

[6] Chen B, Fan VY, Chou Y-J, Kuo C-C (2017). Costs of care at the end of life among elderly patients with chronic kidney disease: patterns and predictors in a nationwide cohort study. BMC Nephrol.

[7] Tonelli M, Riella M (2014). Chronic kidney disease and the aging population. Nephrol Dial Transplant.

[8] Holmes J, Phillips D, Donovan K, Geen J, Williams JD, Phillips AO (2019). Acute kidney injury, age, and socioeconomic deprivation: evaluation of a national data set. Kidney Int Rep.

[9] Ma YC, Zuo L, Chen JH, Luo Q, Yu XQ, Li Y, Xu JS, Huang SM, Wang LN, Huang W, Wang M, Xu GB, Wang HY (2006). Modified glomerular filtration rate estimating equation for Chinese patients with chronic kidney disease. J Am Soc Nephrol.

[10] Wen CP, Cheng TY, Tsai MK, Chang YC, Chan HT, Tsai SP, Chiang PH, Hsu CC, Sung PK, Hsu YH, Wen SF (2008). All-cause mortality attributable to chronic kidney disease: a prospective cohort study based on 462 293 adults in Taiwan. Lancet..

[11] Chen W, Chen WQ, Wang H, Dong XQ, Liu QH, Mao HP, Tan JQ, Lin JX, Zhou FY, Luo N, He HJ, Richard JJ, Zhou SF, Yu XQ (2009). Prevalence and risk factors associated with chronic kidney disease in an adult population from southern China. Nephrol Dial Transplant.

[12] Zhang L, Zhang P, Wang F, Zuo L, Zhou Y, Shi Y, Li G, Jiao S, Liu Z, Liang W, Wang H (2008). Prevalence and factors associated with CKD: a population study from Beijing. Am J Kidney Dis.

[13] Chen W, Liu Q, Wang H, Chen WQ, Johnson RJ, Dong XQ, Li HY, Ba S, Tan JQ, Luo N, Liu T, He HJ, Yu XQ (2011). Prevalence and risk factors of chronic kidney disease: a population study in the Tibetan population. Nephrol Dial Transplant.

[14] Lin B, Shao L, Luo Q, Ou-yang L, Zhou F, Du B, He Q, Wu J, Xu N, Chen J (2014). Prevalence of chronic kidney disease and its association with metabolic diseases: a cross-sectional survey in Zhejiang province, Eastern China. BMC Nephrol.

[15] Liu W, Yu F, Wu YH, Fang XW, Hu WX, Chen J, Zhou RL, Lin XG, Hao WK (2015). A retrospective analysis of kidney function and risk factors by chronic kidney disease epidemiology collaboration (CKD-EPI) equation in elderly Chinese patients. Ren Fail.

[16] Mazidi M, Rezaie P, Covic A, Malyszko J, Rysz J, Kengne AP, Banach M (2017). Telomere attrition, kidney function, and prevalent chronic kidney disease in the United States. Oncotarget..

[17] National chronic kidney disease fact sheet: general information and national estimates on chronic kidney disease in the United States. http://www.cdc.gov/diabetes/pubs/pdf/kidney_Factsheet.pdf. 2010.

[18] Stevens LA, Li S, Wang C, Huang C, Becker BN, Bomback AS, Brown WW, Burrows NR, Jurkovitz CT, McFarlane SI, Norris KC, Shlipak M, Whaley-Connell AT, Chen SC, Bakris GL, McCullough PA (2010). Prevalence of CKD and comorbid illness in elderly patients in the United States: results from the kidney early evaluation program (KEEP). Am J Kidney Dis.

[19] Rothenbacher D, Klenk J, Denkinger M, Karakas M, Nikolaus T, Peter R, Koenig W, for the ActiFE Study Group (2012). Prevalence and determinants of chronic kidney disease in community-dwelling elderly by various estimating equations. BMC Public Health.

[20] Garg AX, Papaioannou A, Ferko N, Campbell G, Clarke JA, Ray JG (2004). Estimating the prevalence of renal insufficiency in seniors requiring long-term care. Kidney Int.

[21] Hemmelgarn BR, Zhang J, Manns BJ, Tonelli M, Larsen E, Ghali WA, Southern DA, McLaughlin K, Mortis G, Culleton BF (2006). Progression of kidney dysfunction in the community-dwelling elderly. Kidney Int.

[22] Arora P, Vasa P, Brenner D, Iglar K, McFarlane P, Morrison H, Badawi A (2013). Prevalence estimates of chronic kidney disease in Canada: results of a nationally representative survey. CMAJ..

[23] Seck SM, Doupa D, Guéye L, Ba I (2014). Chronic kidney disease epidemiology in northern Senegal a cross-sectional study. IJKD..

[24] Carrero JJ, Hecking M, Chesnaye NC, Jager KJ (2018). Sex and gender disparities in the epidemiology and outcomes of chronic kidney disease. Nat Rev Nephrol.

[25] Nitsch D, Felber DD, von EA GJM, Downs SH, Leuenberger P, Tschopp JM, Brndli O, Keller R, Gerbase MW, Probst-Hensch NM, Stutz EZ, Ackermann-Liebrich U, SAPALDIA team (2006). Prevalence of renal impairment and its association with cardiovascular risk factors in a general population: results of the Swiss SAPALDIA study. Nephrol Dial Transplant.

[26] Chadban SJ, Briganti EM, Kerr PG, Dunstan DW, Welborn TA, Zimmet PZ, Atkins RC (2003). Prevalence of kidney damage in Australian adults: the AusDiab kidney study. J Am Soc Nephrol.

[27] Ninomiya T, Kiyohara Y, Kubo M, Tanizaki Y, Doi Y, Okubo K, Wakugawa Y, Hata J, Oishi Y, Shikata K, Yonemoto K, Hirakata H, Iida M (2005). Chronic kidney disease and cardiovascular disease in a general Japanese population: the Hisayama study. Kidney Int.

[28] Viktorsdottir O, Palsson R, Andresdottir MB, Aspelund T, Gudnason V, Indridason OS (2005). Prevalence of chronic kidney disease based on estimated glomerular filtration rate and proteinuria in Icelandic adults. Nephrol Dial Transplant.

[29] Cirillo M, Laurenzi M, Mancini M, Zanchetti A, Lombardi C, De Santo NG (2006). Low glomerular filtration in the population: prevalence, associated disorders, and awareness. Kidney Int.

[30] Silbiger SR, Neugarten J (2003). The role of gender in the progression of renal disease. Adv Ren Replace Ther.

[31] Antonello P, Jennifer BG, Marco M, Doloretta P, Alice A, Maria GP, Liana F, Lenuta B, Nicolò C, Alessandro D, Francesco L, Gonçalo RA, David S, Francesco C (2014). Prevalence of CKD and its relationship to eGFR related genetic loci and clinical risk factors in the SardiNIA study cohort. J Am Soc Nephrol.

[32] McClellan W, Warnock DG, McClure L, Campbell RC, Newsome BB, Howard V, Cushman M, Howard G (2006). Racial differences in the prevalence of chronic kidney disease among participants in the reasons for geographic and racial differences in stroke (REGARDS) cohort study. J Am Soc Nephrol.

[33] Hallan SI, Coresh J, Astor BC, Asberg A, Powe NR, Romundstad S, Hallan HA, Lydersen S, Holmen J (2006). International comparison of the relationship of chronic kidney disease prevalence and ESRD risk. J Am Soc Nephrol.

[34] Weiss JW, Boyd CM (2017). Managing complexity in older patients with CKD. Clin J Am Soc Nephrol.

[35] Jayasumana C, Fonseka S, Fernando A, Jayalath K, Amarasinghe M, Siribaddana S, Gunatilake S, Paranagama P (2015). Phosphate fertilizer is a main source of arsenic in areas affected with chronic kidney disease of unknown etiology in Sri Lanka. Springerplus..

[36] Ciavarella A, Silletti A, Forlani G, Morotti L, Borgnino LC, D’Apote M, Vannini P (1989). A screening test for microalbuminuria in type 1 (insulin-dependent) diabetes. Diabetes Res Clin Pract.

[37] Hemmelgarn BR, Manns BJ, Lloyd A, James MT, Klarenbach S, Quinn RR, Wiebe N, Tonelli M (2010). Relation between kidney function, proteinuria, and adverse outcomes. JAMA..

[38] Lim D, Lee D-Y, Cho SH, Kim OZ, Cho SW, An SK, Kim HW, Moon KH, Lee MH, Kim B (2014). Diagnostic accuracy of urine dipstick for proteinuria in older outpatients. Kidney Res Clin Pract.

[39] Hoerger TJ, Wittenborn JS, Segel JE, Burrows NR, Imai K, Eggers P, Pavkov ME, Jordan R, Hailpern SM, Schoolwerth AC, Williams DE (2010). A health policy model of CKD: 2. The cost-effectiveness of microalbuminuria screening. Am J Kidney Dis.

[40] Wang ZH, Wang LH, Li YC, Zhang M, Hu N, Wang LM (2012). Current status of diabetes, hypertension and dyslipidemia among older Chinese adults in 2010. Chin J Prev Med.

